# Hemodynamic and metabolic changes during hypercapnia with normoxia and hyperoxia using pCASL and TRUST MRI in healthy adults

**DOI:** 10.1177/0271678X211064572

**Published:** 2021-12-01

**Authors:** Pieter T Deckers, Alex A Bhogal, Mathijs BJ Dijsselhof, Carlos C Faraco, Peiying Liu, Hanzhang Lu, Manus J Donahue, Jeroen CW Siero

**Affiliations:** 1 Department of Neurosurgery, University Medical Center Utrecht, Utrecht, Netherlands; 2 Department of Radiology, Center for Image Sciences, University Medical Center Utrecht, Utrecht, Netherlands; 3 Department of Radiology and Nuclear Medicine, Amsterdam Neuroscience, Amsterdam UMC (location VUmc), Amsterdam, Netherlands; 4 Radiology and Radiological Sciences, Vanderbilt University Medical Center, Nashville, Tennessee, USA; 5 Department of Radiology, Johns Hopkins University School of Medicine, Baltimore, Maryland, USA; 6 Spinoza Centre for Neuroimaging, Amsterdam, Netherlands

**Keywords:** Carbogen, cerebral metabolic rate of oxygen, cerebral venous oxygenation, hypercapnia, hyperoxia

## Abstract

Blood oxygenation level-dependent (BOLD) or arterial spin labeling (ASL) MRI with hypercapnic stimuli allow for measuring cerebrovascular reactivity (CVR). Hypercapnic stimuli are also employed in calibrated BOLD functional MRI for quantifying neuronally-evoked changes in cerebral oxygen metabolism (CMRO_2_). It is often assumed that hypercapnic stimuli (with or without hyperoxia) are iso-metabolic; increasing arterial CO_2_ or O_2_ does not affect CMRO_2_. We evaluated the null hypothesis that two common hypercapnic stimuli, ‘CO_2_ in air’ and carbogen, are iso-metabolic. TRUST and ASL MRI were used to measure the cerebral venous oxygenation and cerebral blood flow (CBF), from which the oxygen extraction fraction (OEF) and CMRO_2_ were calculated for room-air, ‘CO_2_ in air’ and carbogen. As expected, CBF significantly increased (9.9% ± 9.3% and 12.1% ± 8.8% for ‘CO_2_ in air’ and carbogen, respectively). CMRO_2_ decreased for ‘CO_2_ in air’ (−13.4% ± 13.0%, p < 0.01) compared to room-air, while the CMRO_2_ during carbogen did not significantly change. Our findings indicate that ‘CO_2_ in air’ is not iso-metabolic, while carbogen appears to elicit a mixed effect; the CMRO_2_ reduction during hypercapnia is mitigated when including hyperoxia. These findings can be important for interpreting measurements using hypercapnic or hypercapnic-hyperoxic (carbogen) stimuli.

## Introduction

Advanced MRI techniques provide an avenue to image functional parameters such as oxygen extraction fraction (OEF), cerebral blood flow (CBF), cerebral blood volume (CBV), and the cerebral metabolic rate of oxygen consumption (CMRO_2_). Thereby, these MRI techniques can provide metabolic and hemodynamic metrics similar to those obtained using PET, but non-invasively and at higher spatial and temporal resolution. One such approach is calibrated fMRI (or calibrated BOLD) that can be used to scale task-related BOLD fMRI responses to the underlying CMRO_2_ changes.^1^ This technique has found applications to examine metabolic demands associated with functional tasks but also ageing and brain disease.^2–5^

The BOLD calibration models rely on measurements of both BOLD and CBF signal changes during either hypercapnic respiratory challenges or a combination of hypercapnic-hyperoxic challenges.^6–9^ Modulations in arterial gas tensions evoke predictable physiological responses that are used to contextualize fMRI signal changes.^10–13^ In short, hypercapnia is a potent vasodilator that leads to significant increases in CBV, CBF, and accompanying increases in venous blood oxygenation, while hyperoxia is thought to only modulate the blood oxygenation through plasma-dissolved O_2_ and increased hemoglobin (Hb) bound O_2_. This dual-action convolutes the net hemodynamic and metabolic response through its influence on the oxygen saturation curve via the Bohr effect (i.e. how CO_2_ affects the binding affinity of Hb for O_2_) and the Haldane effect (i.e. how O_2_ affects the binding affinity of Hb for CO_2_). An original assumption during the early development of the calibration model was that increasing arterial levels of CO_2_ and O_2_ had a negligible effect on neuronal metabolism and thus CMRO_2_.^4^ This notion has since been challenged, and correction methods have been devised to account for the possibility that changing arterial O_2_ or CO_2_ tensions do indeed modulate neuronal functioning and CMRO_2_^9,11–18^ Previous simulation work on calibrated BOLD models has reported that for non iso-metabolic hypercapnic challenges for calibration, one can find a significant error in estimating the basal OEF and activation-induced CMRO_2_ changes in calibrated BOLD studies.^10,19,20^ Correction methods for situations in which arterial O_2_ and CO_2_ tensions change simultaneously have not yet been adopted.

Quantitative assessment of venous oxygenation (Y_v_), while considering the combined effects of changing CBF and OEF, provides an avenue to examine the iso-metabolic assumptions associated with respiratory stimuli via inferred changes in CMRO_2_. This can be performed non-invasively using a technique known as T_2_ -Relaxation-Under-Spin-Tagging (TRUST) MRI.^21^ Direct knowledge of the effects that changing arterial O_2_ and CO_2_ tensions have on CMRO_2_ has widespread implications. For instance, appropriate corrections can improve the accuracy of fMRI techniques for neuroscientific applications, refine BOLD signal models that simulate magnetic susceptibility effects under different physiological conditions,^22–24^ and improve the interpretation of CVR for clinical applications via a more robust understanding of concomitant CMRO_2_ changes.^25–28^

The use of respiratory challenges during MRI in various patient populations is becoming more widespread. In many of these cases, the pathophysiology of neurological and cerebrovascular disease leads to altered cerebral metabolism. With this in mind, it becomes essential to identify co-factors that influence cerebral metabolism and are accounted for accordingly. Therefore, this study aimed to evaluate the assumption that hypercapnic stimuli applied in healthy adults are iso-metabolic. To achieve this, we used ASL and TRUST MRI for measures of global CBF and Y_v_ to compare measures of CMRO_2_ under two commonly used hypercapnic conditions (‘CO_2_ in air’: 5% CO_2_ + 21% O_2_ + 74% N_2_; and carbogen: 5% CO_2_ and 95% O_2_). The CMRO_2_ value for each condition was calculated and compared with the CMRO_2_ value obtained during the breathing of medical air (referred to as ‘room-air’).

## Methods

### Volunteer demographics

Healthy volunteers (n = 10; 3 F/7 M; age = 29.4 ± 3.4 years) provided informed, written consent following the ethical standards of the Vanderbilt University Institutional Review Board, the Vanderbilt University Human Research Protection Program, as well as with the Helsinki Declaration of 1975 (and as revised in 1983). All components of this study were performed in compliance with the Health Insurance Portability and Accountability Act. All study components were reviewed and approved by the local Institutional Review Board (IRB Study 111116).

### MRI experiment

**Figure 1.**
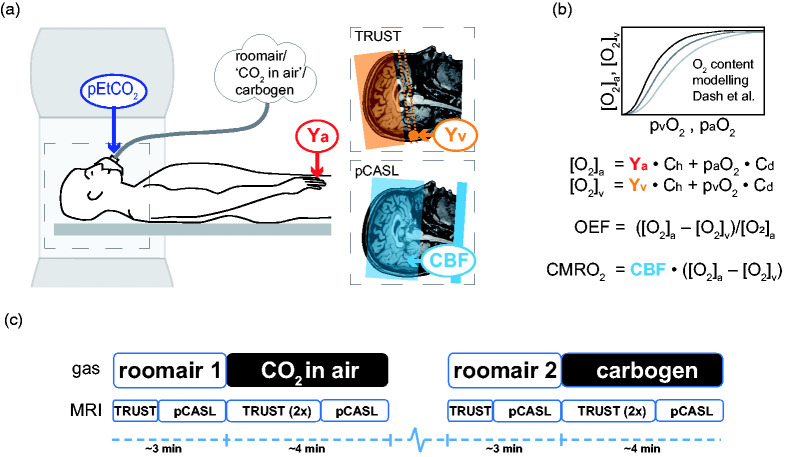
Schematic overview of the experimental design. a) the subject in the MRI with the three different inspired gases and the physiological measurements of pEtCO_2_ (purple) from the breathing mask, arterial oxygenation Y_a_ (red) from a pulse-oximeter, venous oxygenation Y_v_ (orange), and CBF (blue). Conceptual planning for cerebral venous oxygenation (Y_v_) using T2-relaxation-underspin-tagging (TRUST) MRI is depicted by the orange region, where the dotted lines represent the measurement plane and the circle the location of the occipital part of the superior sagittal sinus. Whole-brain CBF values were acquired using a pseudo-continuous arterial spin labeling pCASL sequence (planning depicted in blue). b) The [O_2_]_a_, [O_2_]_v_, p_a_O_2,_ and p_v_O_2_ values were calculated using a physiological blood oxygen content model (Dash et al.) and were applied for computing CMRO_2_ and OEF using the formulas shown. c) Experimental design of the gas delivery paradigm. The dashed blue line represents the rest period (∼30 s) given to allow pEtCO_2_ and Y_v_ to equilibrate. The order of ‘CO_2_ in air’ and carbogen conditions and the TRUST and pCASL scan was randomized between subjects.

All MRI measurements were performed at 3.0 T (Philips Healthcare, Best, The Netherlands). For a schematic overview of the MRI experiment see Figure 1. Participants were fitted with a nasal cannula (Salter Labs, Arvin, CA, USA, no. 4000) to monitor the end-tidal partial pressure of CO_2_ (pEtCO_2_) and a custom non-rebreathing face mask (Salter Labs, no. 8005) for gas stimulus administration. The masks were close-fitting and covered the nose and mouth. Elastic straps were used to reduce leakage. Gases were delivered from compressed cylinders outside the scan room and administered at 12 L/min. This flow rate was optimized in preliminary studies and was found to provide sufficient gas delivery in the presence of potential small leaks in the mask, while also maintaining comfort. Physiological monitoring was performed using an Invivo Research (Gainesville, FL, USA, 3150 MRI) monitor and a remote monitor (Millenia Vital System, Gainsville, FL, USA, 3115 MVS). Monitored parameters included partial pressure of end-tidal CO_2_ (pEtCO_2_ in mmHg), and peripheral arterial oxygenation (Y_a_). The repeatability of this setup has been reported previously^29^ and a similar setup is used in a previously reported work.^24^

Venous oxygenation (Y_v_) MRI measurements were performed on the occipital part of the superior sagittal sinus using TRUST MRI (TR = 3s, TI = 1.2s, voxel size = 3.4 × 3.4 × 5 mm^3^, four T_2_ weightings (effective TEs: 0, 40, 80, and 160 ms), with a τ_CPMG_ = 10 ms, and 3 averages per T_2_ weighting yielding a total scan duration of 1 min 12 s.^30,31^ CBF measurements were performed using pCASL MRI with a multi-slice echo-planar imaging readout. Acquisition parameters were post-label delay (PLD) = 1.7 s, label duration =1.5 s, TR/TE = 3900/13.1 ms, measurements = 13, field-of-view = 240 × 240 × 119 mm^3^, spatial resolution = 3 × 3 × 7 mm^3^, slices = 17, SENSE factor = 1.8. The TRUST scan was repeated once for the hypercapnic breathing conditions but was only performed once for the room-air conditions. TRUST and pCASL data were sequentially acquired throughout the following paradigm: 3 min room-air (‘room-air 1’) – ∼4 min of 5% CO_2_ balanced with medical-grade atmospheric air (5% CO_2_/21% O_2_/74% N_2_; ‘CO_2_ in air’: as described earlier^24^) – 3 min room-air (‘room-air 2’) – ∼4 min carbogen (5% CO_2_, 95% CO_2_; Figure 1). For each hypercapnic breathing condition, the two Y_v_ values were first estimated from the two TRUST measurements and subsequently averaged into a single Y_v_ value per condition. In addition, a calibration M_0_ scan was acquired for both groups for CBF quantification, using identical acquisition geometry as the pCASL scan, but with TR = 15 s, and the spin labeling pulse train turned off. The order of ‘CO_2_ in air’ and carbogen and the TRUST and pCASL scan was randomized between subjects and time (∼30 s) was allowed for pEtCO_2_ and Y_v_ to equilibrate between the two hypercarbic conditions.

### Analysis

#### CBF quantification

CBF quantification was performed using FSL BASIL;^32^ pCASL label and control images were pair-wise subtracted, and a single-compartment kinetic model was applied to the data using tissue-blood partition coefficient of water λ = 0.9 ml/g and pCASL labeling efficiency α = 0.85.^33^ Slice time correction was incorporated by using slice-specific PLD values. For equilibrium blood water magnetization, the calibration M_0_ image was used. An arterial blood water T_1_ (T_1a_) reduction from 1.65 s (used for the normoxic conditions) to 1.49 s (hyperoxic conditions) was used to account for hyperoxia-dependent changes related to the carbogen condition, as reported previously.^34^

For tissue T_1_ and arterial (bolus) arrival time, gray matter values (GM) were used in BASIL FSL, 1.3 s^35^ and 1.3 s^29,36^ for pCASL, respectively, including a GM arrival time reduction of 5% for the hypercapnic conditions.^29,36^ Global CBF was estimated using subject-specific brain tissue segmentation masks and associated tissue probability maps of GM and white matter (WM). We focused on global CBF as opposed to solely GM CBF as the TRUST measurement for Y_v_(%) also samples a global value. Tissue segmentation was performed on the M_0_ image that exhibited adequate GM and WM contrast using FSL FAST.^37^ The global CBF estimation excluded subarachnoidal space and ventricular cerebral spinal fluid (CSF), cerebellum and brainstem regions. CSF regions were removed using the subject-specific segmentations masks from FSL FAST, the cerebellum and brainstem regions were removed using the MNI Structural Atlas and the Harvard-Oxford Structural Atlas^38^ MNI based (Montreal Neurological Institute) ROIs, respectively, available in FSL.^39^ Segmentation and atlas ROIs were constrained by the subject’s whole-brain mask obtained from the M_0_ image using FSL BET.^40^ Note that GM values were used for the tissue T_1_ and arterial arrival time for all voxels using FSL BASIL for the CBF quantification. However, these values are not appropriate for WM CBF included in this study to obtain global CBF and can lead to an underestimation for WM CBF (See Supplementary Material Figure 1). We computed a correction factor for WM CBF using the WM tissue T_1_ and arterial arrival time, 0.84 s^35^ and 1.7 s,^36^ respectively, as recently reported by Juttakonda et al.^36^ (see Supplementary Material for computation). Average global CBF was recomputed by summing the GM and WM CBF maps weighted by the tissue probability maps and the WM correction factor for WM (∼1.20, see Supplementary Material and Supplementary Figure 1). Voxels with outlier CBF values were discarded for absolute CBF values larger than two standard deviations above the mean global CBF, i.e. |CBF_voxel_| > mean (global CBF) ± 2 std (global CBF). To visualize the group average CBF map results, the subjects’ CBF maps were first spatially normalized to the standard MNI 2 mm^3^ stereotaxic space using FSL FLIRT with 12 degrees of freedom affine registration and sinc interpolation.^41^ The subject averaged pCASL control image was used as an intermediate step.

#### CMRO2 and OEF estimation

Calculation of Y_v_ from the TRUST MRI data was done using the method described previously.^13^ For the repeated scans, i.e. for the ‘CO_2_ in air’ and ‘carbogen’ conditions, the fitted Y_v_ values were averaged. To account for the plasma dissolved O_2_ present during the hyperoxic condition, we computed the total arterial O_2_ content ([O_2_]_a_), venous O_2_ content ([O_2_]_v_), and partial pressure of venous O_2_ (p_v_O_2_) using the physiological model by Dash et al.^42^ that models the blood O_2_ and CO_2_ content by generating the hemoglobin-O_2_ and CO_2_ dissociation (saturation) curve and computing the plasma-dissolved O_2_^24,42^ The model takes into account the Bohr effect for which O_2_ binding to hemoglobin is inversely related to the presence of CO_2_ by using the measured pEtCO_2_ (see Table 1). This will yield subject-specific and breathing condition-specific hemoglobin O_2_ saturation curves, dependent on blood pO_2_ and pCO_2_. We will show the O_2_ saturation curve by Dash et al.^42^ and the commonly used saturation curve by Severinghaus (1), that depends only on the pO_2_:^43^

(1) $$Y_{\left(\%\right)}=\left(\frac{1}{\frac{1}{pO_{2}^{3}+150\cdot pO_{2}}\cdot 23400+1}\right)\cdot 100\%$$

**Table 1.** Group average cerebral blood flow (CBF), venous oxygenation (Y_v_), oxygen extraction fraction (OEF), and cerebral metabolic rate of oxygen (CMRO_2_) results for the room-air, ‘CO_2_ in air’ and carbogen breathing conditions. The group average change in end-tidal CO_2_ (pEtCO_2_), global CBF, Y_v_, OEF, and CMRO_2_ are also shown.

	end-tidal CO2	cerebral blood flow			venous oxygenation		oxygen extraction fraction		cerebral metabolic rate of O2	
condition	ΔpEtCO_2_ (mmHg)	CBF (ml/100g/min)	ΔCBF (ml/100g/min)	ΔCBF (%)	Y_v_ (%)	ΔY_v_ (%)^a^	OEF	ΔOEF (%)	CMRO_2_ (µmol/100g/min)	ΔCMRO_2_ (%)^b^
room-air 1		54.9 ± 8.2			67.3 ± 3.6		0.3 ± 0.0		144.5 ± 25.5	
room-air 2		52.3 ± 6.8			65.3 ± 5.1		0.3 ± 0.0		147.8 ± 33.3	
CO_2_ in air	4.2 ± 1.7***	61.0 ± 8.0	6.1 ± 5.4*	9.9 ± 9.3*	73.9 ± 4.7	8.8 ± 3.2***	0.2 ± 0.1	-30.8 ± 15.2***	123.9 ± 24.8	−13.4 ± 13.0*
carbogen	4.5 ± 2.2***	60.0 ± 9.1	7.7 ± 5.4**	12.1 ± 8.8**	78.6 ± 7.3	16.3 ± 8.9***	0.3 ± 0.1	-39.6 ± 52.2*	139.3 ± 34.1	−2.0 ± 27.0^n^

Values represent the group average ± standard deviation.

^a^This is the fractional change in percentage in Y_v_, i.e. not percentage points.

**Figure 2.**
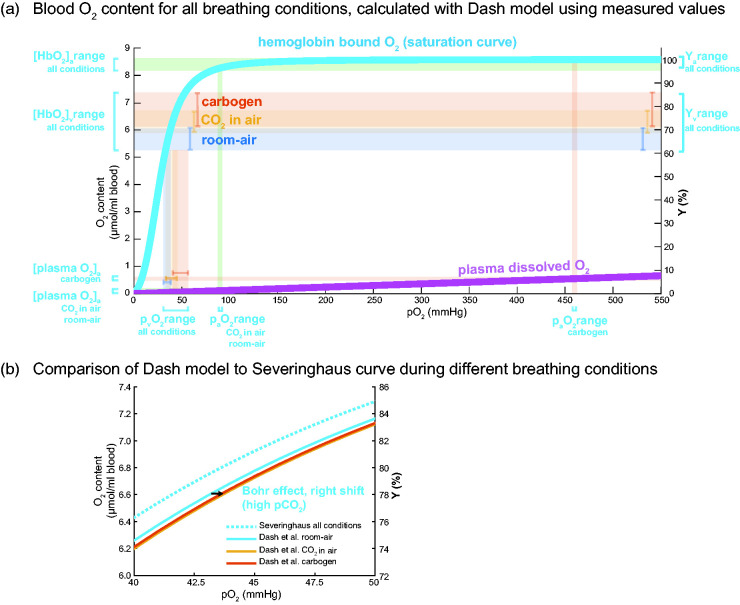
Oxygen saturation and content in blood for the different breathing conditions. The measured pEtCO_2_ values and assumed hematocrit values were used as input for the Dash et al. model to generate the subject-specific curves on saturation, hemoglobin bound and plasma dissolved O_2_ content over a range of pO_2_ between 0 and 550 mmHg. a) Group average hemoglobin bound O_2_ saturation curve (light blue) as computed using Dash et al. physiological model ^42^ and the plasma dissolved O_2_ curve (magenta) as a function of the partial pressure of O_2_ (pO_2_). On the right y-axis, the measured ranges of hemoglobin blood oxygenation Y_a_(%) and Y_v_(%) for the different breathing conditions (room-air in blue, ‘CO_2_ in air’ in orange, carbogen in red, and the corresponding O_2_ content in μmol/ml blood on the left y-axis ([HbO_2_]_a_ and [HbO_2_]_v_ ranges). The associated partial pressure ranges of O_2_ (p_v_O_2_ and p_a_O_2_) found via the O_2_ saturation curve (light blue) are shown on the x-axis. Note the high p_a_O_2_ for the carbogen condition and the associated plasma dissolved O_2_ content shown on the bottom left (red). b) A zoomed part of the O_2_ saturation curve (light blue in a)) showing the traditional O_2_ saturation curve by Severinghaus (dotted light-blue) compared to the revised model by Dash et al. with dependency on the subject’s pCO_2_ and Hct. The hypercapnic conditions induce a right shift caused by the Bohr effect, shown by the arrow (‘CO_2_ in air’ in orange, carbogen in red). The effect of this right shift, however, on the arteriovenous O_2_ difference is negligible for all breathing conditions. See Figure 3 for the O_2_ content and the arteriovenous difference values (boxplots) for all breathing conditions.

^b^When ignoring the venous plasma dissolved O_2_, we find a ΔCMRO_2_(%) of −11.30% and 3.11% for ‘CO_2_ in air’ and carbogen, respectively, i.e. showing a similar change. When also ignoring the arterial plasma dissolved O_2_, we find a ΔCMRO_2_(%) of −12.06% and −22.30% for ‘CO_2_ in air’ and carbogen, respectively, showing a substantial difference for the carbogen condition. Note, when using the commonly used Severinghaus O_2_ saturation curve instead of the model by Dash et al.^42^ (see Figure 2(b)), we find a ΔCMRO_2_(%) of -9.33% and 0.35% for ‘CO_2_ in air’ and carbogen, respectively, also showing a similar change.

*p-value <0.05 significant change found (Student’s T-test) with respect to the preceding room-air condition, **p-value <0.005, ***p-value <0.001, ^n^no significant difference found.

The partial pressure of alveolar O_2_ (p_A_O_2_) was assumed 104 mmHg respectively for the ‘CO_2_ in air’ and room-air conditions.^44^ For these normoxic conditions, alveolar, p_A_O_2_, was converted to arterial O_2_ pressure (p_a_O_2_) using the alveolar-arterial O_2_ pressure gradient, which depends on the p_A_O_2_ and age.^12^ For the carbogen condition, we used the values from previous reports on direct measurements of arterial partial pressure PaO_2_ for carbogen in healthy subjects that used an arterial line during PET examination:

p_a_O_2,carbogen_ = 460 mmHg.^45,46^ The venous partial pressure of CO_2_ (p_V_CO_2_) was offset by +5 mmHg compared to the measured pEtCO_2_.^44^ The p_v_CO_2_ estimates are needed to incorporate the Bohr effect; when locally CO_2_ partial pressure is increased, the hemoglobin affinity for O_2_ is decreased, which we can expect in peripheral and cerebral tissue and under hypercapnic conditions resulting in a right shift of the HbO_2_ curve. The mathematical model put forward by Dash et al.^42^ incorporates the p_v_CO_2_ in the K_Hb_O_2_ factor, the apparent equilibrium constant for binding O_2_ to hemoglobin. The K_Hb_O_2_ factor itself is a complex equation describing the binding kinematics of O_2_ to hemoglobin (see equations 1a,b and 3a,b in Dash et al.^42^) Note that changes in pH, temperature, and 2,3 -DPG will also shift the HbO_2_ curve and can in principle be incorporated into the physiological model. Hematocrit (Hct) values of 0.42 for males and 0.4 for females were assumed.

OEF and CMRO_2_ were computed using formulas (2) and (3) using equations (4) and (5) for the calculation of [O_2_]_a_ and [O_2_]_v_, i.e. the sum of the hemoglobin bound O_2_ and plasma dissolved O_2_ content.

(2) $$\text{OEF}=\frac{{\left[O_{2}\right]}_{a}–{\left[O_{2}\right]}_{v}}{{\left[O_{2}\right]}_{a}}$$

(3) $$\text{CMR}{\text{O}}_{\text{2}}=\text{CBF}\cdot ({\left[O_{2}\right]}_{a}–{\left[O_{2}\right]}_{v})$$

(4) with$${\left[O_{2}\right]}_{a}=Y_{a}\cdot C_{h}+p_{a}O_{2}\cdot C_{d}$$

(5) and$${\left[O_{2}\right]}_{v}=Y_{v}\cdot C_{h}+p_{v}O_{2}\cdot C_{d}$$

Constants C_h_ (912 μmol O_2_/100 ml blood for a Hct of 0.45, but adjusted for the assumed Hct values for male and female subjects^42^) and C_d_ (0.138 μmol O_2_/100 ml blood/mmHg p_a_O_2_) are the hemoglobin O_2_ carrying capacity and blood plasma O_2_ dissolving capacity, respectively.^13^ Dash et al. 2016 model^42^ can be downloaded (JSIM and Matlab code) from the NSR Physiome Project repository that contains integrative and descriptive models on human physiology: www.imagwiki.nibib.nih.gov/physiome/jsim/models/webmodel/NSR/SHbO2CO2Dash2016/.

#### Effect of arterial blood water T1a on CBF and CMRO_2_ quantification

The choice of arterial blood water T_1a_ can have a considerable impact on the absolute CBF and CMRO_2_ quantification, and especially during hyperoxic conditions where substantial T_1a_ changes can be expected.^34^ Therefore, we performed a sensitivity analysis to investigate the influence of varying T_1a_ on CBF and CMRO_2_ quantification, caused by for instance hyperoxia but also Hct variations (see Supplementary Figure 2).

In addition, we investigated whether different T_1a_ scenarios impacted the CBF, ΔCBF, and importantly the CMRO_2_ and ΔCMRO_2_ quantification, using commonly used values at 3T for normoxic (T_1,NO_) and hyperoxic (T_1,HO_) conditions. For the normoxic conditions, T_1a_ values were used as reported by Lu et al.^47^ (T_1,NO_ = 1.65s, bovine blood, recommended ASL Whitepaper value^48^) by Pilkinton et al.^49^ (T_1,NO_ = 1.669s, rat blood) commonly used in calibrated BOLD studies,^50^ and the recently modelled and measured value by Li et al.^51,52^ for human blood (T_1,NO_ = 1.898s, human blood).

For the hyperoxic condition (assuming a p_a_O_2_ = 460 mmHg for carbogen), we used the values as reported by Siero et al.,^34^ T_1,HO_ = 1.49s based on T_1,NO_ = 1.65 s (Scenario I), the reported hyperoxic T_1a_ relativity by Ma et al.,^53^ yielding a T_1,HO_ = 1.472s based on T_1,NO_ = 1.65 s (Scenario II), the report by Pilkinton et al.,^49^ T_1,HO_ = 1.527s based on T_1,NO_ = 1.669 s (Scenario III), and the model by Li et al.^51^ where we incorporated the hyperoxic relaxivity by Ma et al.,^53^ yielding a T_1,HO_ = 1.743s based on T_1,NO_ = 1.898 s by Li et al.^51^ (Scenario IV). In summary, four scenarios with different T_1,NO_ and T_1,HO_ combinations were used to assess the effect on the CBF, ΔCBF, CMRO_2_ and ΔCMRO_2_ quantification (see Supplementary Figure 3).

#### Statistical analysis

Statistical analysis was performed to assess significant differences in the change in hemodynamic parameters for each separate hypercapnic condition (‘CO_2_ in air’ versus preceding room-air and carbogen versus preceding room-air) using Student’s T-test in SPSS Statistics version 26 (IBM Business Analytics, New York, USA). The distribution of the data was visually checked with histograms and Q-Q plots, and formally with the Kolmogorov-Smirnov test. A p-value <0.05 was considered significant.

## Results

All ten subjects completed the study with no reported adverse effects. All data were normally distributed. The pEtCO_2_ averaged across subjects increased significantly (p < 0.001) from the ‘room-air 1’ (45.0 ± 3.6 mmHg) to the ‘CO_2_ in air’ conditions (49.2 ± 2.7 mmHg) and from the ‘room-air 2’ (43.7 ± 3.6 mmHg) to the carbogen (48.1 ± 3.0 mmHg) conditions. The change in pEtCO_2_ (ΔpEtCO_2_) is shown in Table 1 for all subjects. Arterial oxygenation (Y_a_) for ‘room-air 1’, ‘room-air 2’, ‘CO_2_ in air’ and ‘carbogen’ conditions were 98.2 ± 0.6%, 98.4 ± 0.4%, 98.5 ± 0.6% and 98.9 ± 0.4%, respectively. Relative to the corresponding room-air conditions, Y_a_ increased less for ‘CO_2_ in air’ than for the carbogen (p = 0.01) condition.

**Figure 3.**
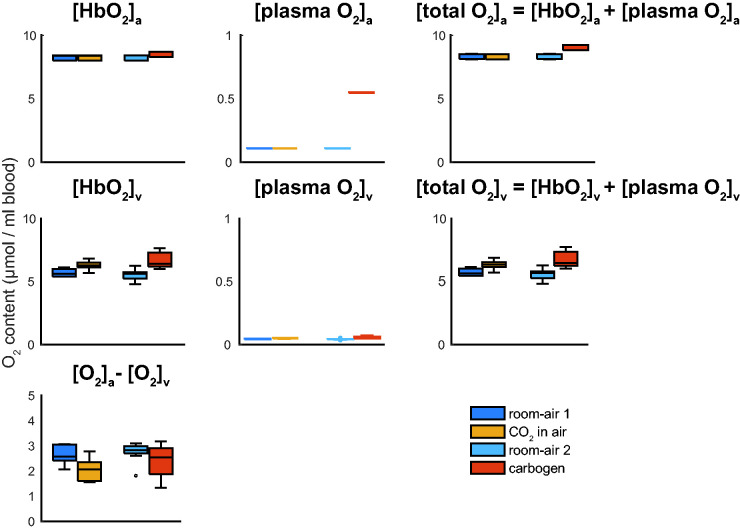
Boxplots showing the group average O_2_ content in μmol per ml blood for hemoglobin bound O_2_ and plasma dissolved O_2_ for the arterial and venous blood respectively, and the arteriovenous difference in O_2_ content needed to compute the CMRO_2_; [HbO_2_]_a_ and [HbO_2_]_v_, [plasma O_2_]_a_, and [plasma O_2_]_v_, the total blood O_2_ content [O_2_]_a_ and [O_2_]_v_, and [O_2_]_a_-[O_2_]_v_ for the different breathing conditions. Noticeable is the increased venous hemoglobin bound O_2_ ([HbO_2_]_v_) for the hypercapnic conditions and the much-increased plasma dissolved O_2_ (arterial, [plasma O_2_]_a_) content for the carbogen condition. Also, note the much smaller y-axis scale for the plasma dissolved O_2_ content, showing that the venous plasma dissolved O_2_ plays a negligible role in the arteriovenous difference to compute CMRO_2_ for all breathing conditions. The boxplots show the minimum, maximum, median and interquartile range, open circles denote outliers.

Figure 2(a)

shows the hemoglobin bound O_2_ curve and the plasma dissolved O_2_ curve as a function of the partial pressure of O_2_ (pO_2_). These curves were used to show the range of HbO_2_ and plasma O_2_, for both the arterial and venous networks, using the subject-specific Y_v_ and P_a_O_2_ values for all three breathing conditions. The curves were computed using the Dash et al.^42^ physiological model, using the (assumed) Hct and measured pEtCO_2_ as the input for each subject, and then averaged across subjects. Note the high p_a_O_2_ for the carbogen condition and the associated plasma dissolved O_2_ content; venous plasma dissolved O_2_ plays a negligible role in the arteriovenous difference to compute CMRO_2_. Figure 2(b) depicts a selected portion of the O_2_ saturation curve derived from the Severinghaus equation (dotted light-blue), and the model by Dash et al.^42^ that incorporates the dependencies on pCO_2_ and Hct, as well as the ‘right shift’ that occurs under hypercapnia due to the Bohr effect. Note that the effect of the latter is negligible for the arteriovenous O_2_ difference for all hypercapnic breathing conditions. In addition, the effect of hyperoxia during carbogen inhalation did not notably increase the venous blood CO_2_ content through the Haldane effect (<1%, data not shown). The blood CO_2_ content is mostly in the form of bicarbonate and an order of magnitude less as dissolved CO_2_ and hemoglobin bound CO_2_. See Figure 3 for the values of the blood O_2_ content and the arteriovenous difference for all breathing conditions.

**Figure 4.**
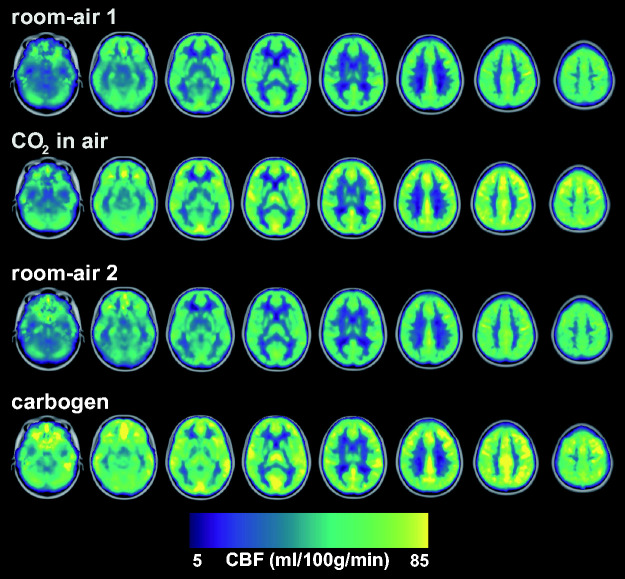
Group average CBF maps for the different conditions where a notable and similar increase in CBF is observed for ‘CO_2_ in air’ and carbogen (see also Table 1). The individual maps were registered to MNI space before averaging and are overlaid on the 2 mm MNI brain template.

**Figure 5.**
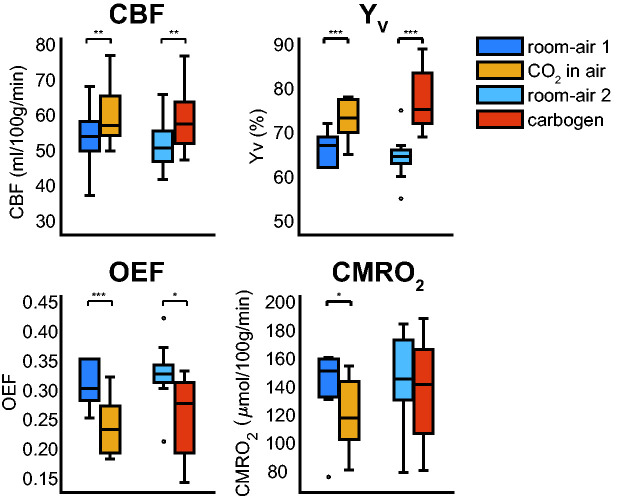
Boxplots showing the group average global CBF, venous oxygenation (Y_v_), computed oxygen extraction fraction (OEF), and cerebral metabolic rate of oxygen (CMRO_2_) for the different conditions. Notable increases in CBF and Y_v_ are observed for both the ‘CO_2_ in air’ and carbogen conditions, with a more considerable Y_v_ increase for the carbogen condition, as expected. A similar reduction in OEF is seen for both conditions. Only significant CMRO_2_ changes are observed for the ‘CO_2_ in air’ condition. The CBF increase for the ‘CO_2_ in air’ and carbogen conditions was not significantly different. *p-value <0.05 significant change found (Student’s T-test) with respect to the preceding room-air condition, **p-value <0.005, ***p-value <0.001. The boxplots show the minimum, maximum, median and interquartile range, open circles denote outliers.

The group average global CBF maps showed similar increases in CBF during the ‘CO_2_ in air’ and carbogen conditions compared to room-air (Figure 4). Significant changes in global CBF, Y_v_ and OEF for ‘CO_2_ in air’ and carbogen were observed (Figure 5, Table 1). The percentage CBF increase for ‘CO_2_ in air’ and carbogen was 9.9 ± 9.3% and 12.1 ± 8.8%, respectively. The absolute CBF increase per mmHg pEtCO_2_ for ‘CO_2_ in air’ and carbogen were 1.7 ± 1.5 and 2.8 ± 3.2 ml/100g/min/mmHg, respectively. Relative and absolute CBF changes between each hypercapnic condition were not statistically significant (Table 1). Compared to the room-air condition, venous oxygenation Y_v_ increased by 8.8 ± 3.2% (p < 0.001) for the ‘CO_2_ in air’ condition. As expected, a more considerable increase in Y_v_ was observed for the carbogen condition (16.3 ± 8.9%, p < 0.001) due to the hyperoxic gas mixture in addition to the increased blood flow. No significant differences in OEF were found between ‘CO_2_ in air’ and carbogen conditions; the reduction in OEF was 30.8 ± 15.2% (p < 0.0001) and 39.6 ± 52.2% (p < 0.05) respectively compared to room-air (see Table 1). Note that the amount of plasma dissolved O_2_ was included in the computation of the OEF and CMRO_2_ for both conditions (see Methods).

Significant CMRO_2_ changes were only observed for the ‘CO_2_ in air’ condition, showing a decrease of 13.4 ± 13.0% (p < 0.01) (see Table 1). ΔCMRO_2_ did not significantly change for the carbogen condition, −2.0 ± 27.0%. Note, when using the commonly used Severinghaus O_2_ saturation curve instead of the model by Dash et al.^42^ (see Figure 2(b)), we find a ΔCMRO_2_(%) of -10.4% and 0.3% for ‘CO_2_ in air’ and carbogen, respectively, also showing a similar change. When ignoring the venous plasma dissolved O_2_ in the CMRO_2_ computation, the estimated ΔCMRO_2_(%) were -12.7% and -1.3% for ‘CO_2_ in air’ and carbogen, respectively. When also ignoring the arterial plasma dissolved O_2_, ΔCMRO_2_(%) was -13.8% and -20.6% for ‘CO_2_ in air’ and carbogen, respectively. Values for all subjects are shown in Supplementary Tables 1 and 2. We performed a sensitivity analysis of variations in arterial blood water T_1a_ on CBF and CMRO_2_ quantification caused by, for instance, hyperoxia and Hct variations. An increase in the hyperoxic T_1,HO_ value of 5% from the reference T_1,HO_ (=1.49 ms) leads to a decrease of CBF and CMRO_2_ of ∼4.0%, while a T_1,HO_ decrease of 5% leads to an increase of CBF and CMRO_2_ of ∼4.7% (Supplementary Figure 2). We also investigated whether different T_1a_ scenarios impacted the results using commonly used and recently reported T_1,NO_ and T_1,HO_ values. The absolute CBF and CMRO_2_ results change in value as expected, however, the ΔCBF and notably the ΔCMRO_2_ changes did not change significantly for the different T_1_ scenarios (Supplementary Figure 3).

## Discussion

### Main findings

Using a combination of quantitative CBF and Y_v_ measurements along with physiological modeling based on measured arterial blood gas values, our results reinforce the notion that hypercapnia is not an iso-metabolic stimulus. Our main findings were threefold: 1) global CMRO_2_ reduces with normoxic-hypercapnia; 2) correction for changes in the oxygen saturation curve due to hypercapnia did not significantly affect the calculation of global CMRO_2_; 3) inhalation of carbogen gas appears to elicit a mixed effect where the reduction in CMRO_2_ seen during normoxic-hypercapnia is mitigated with the inclusion of a hyperoxic stimulus.

Our results showed that the inspiration of 5% CO_2_ in room-air led to an average decrease in global CMRO_2_ of approximately 13.4%. This falls directly in line with the 13.4% reduction reported by Xu et al.^12^ Similar findings have been reported by Thesen et al.,^18^ who measured magnetoencephalogram responses while breathing air and a 5% CO_2_ mixture and showed clear decreases in event-related field potentials under hypercapnia.^18^ Also, for non-human primates, a clear reduction of CMRO_2_ with hypercapnia has been reported.^14,16^

The notion that CMRO_2_ reductions follow a linear behavior for increasing levels of arterial CO_2_ forms the basis of the updated BOLD calibration model reported by Driver et al.^11^ Several reports, including those by Jain et al.^54^ and Chen et al.,^55^ showed no significant difference in CMRO_2_ under hypercapnia. Our measured OEF values (0.32 for room-air, 0.25 for ‘CO_2_ in air’, and 0.26 for carbogen) were in line with previous work reported in healthy subjects using ASL^56^ and PET.^46^ The decrease in OEF for the CO_2_ condition can be explained by the decrease in CMRO_2_ and increase in CBF, while the decrease in OEF for the carbogen condition may be (partly) attributed to the higher oxygen content in the arterial blood and the increase in CBF.

The changes in end-tidal CO_2_ we observed during ‘CO_2_ in air’ (∼4.2 mmHg) were lower than the 8-10mmHg changes typically observed in healthy subjects using a similar stimulus. Lower values may be attributed to potential gas leaking from the masks, leading to less efficient gas administration. The average baseline pEtCO_2_ value measured in our subjects during the room-air condition was 44.3 ± 3.5 mmHg. These values are higher than the ∼40mmHg typically quoted in physiology textbooks and higher than what was reported by Chen et al.,^55^ and Jain et al.^54^ in healthy subjects. The elevated baseline CO_2_ in our experiments likely stems from the fact that we used a facemask and did not clamp end-tidal gas values. This may have effectively increased dead-space, leading to more rebreathing and increased pEtCO_2_ as has been shown recently for the use of surgical facemasks during MRI acquisition.^57^ The facemask use and its effect on dead-space may also have contributed to higher room-air Y_v_ values in our subjects. Our reported Y_v_ during the two room-air conditions, 67.3 ± 3.6%, and 65.3 ± 5.1%, however, are in line with Jain et al.^54^ but are higher than Chen et al.^55^ It should be noted that both studies reported higher increases in CBF during hypercapnia, which may account for the differences in the estimated CMRO_2_ change. The elevated room-air pEtCO_2_ that we measured could have mitigated subsequent increases in pEtCO_2_ due to a reduced alveolar-arterial CO_2_ gradient during the CO_2_ stimulus. For pre-dilated baseline states (due to CO_2_ buildup in the mask), it is conceivable that further increases in arterial CO_2_ may push both absolute CBF^58,59^ and associated BOLD^25^ signals when considering calibrated MRI beyond the known linear response regime. We measured an increase in CBF of approximately 1.7 ± 1.5 and 2.8 ± 3.2 ml/100g/min per mmHg change in pEtCO_2_ during the ‘CO_2_ in air’ and carbogen conditions, respectively. These increases are in line with results observed for both carbogen (5% O_2_ and 95% CO_2_) and (5%) CO_2_-enriched air inhalation in healthy volunteers.^60^

When measuring changes in physiological parameters in response to external stimuli, the accuracy of the stimulus delivery as well as the measurement of CBF and Y_v_ is an important concern. Particularly since the data interpretation relies on physiological modeling, our methods could have benefitted from tighter control and targeting of arterial blood gases via the use of a computer-controlled gas delivery system. This could provide more accurate measurements of end-tidal O_2_ for more accurate estimates of blood O_2_ content using physiological modeling by Dash et al.^42^ Also, there is the notion of adequate sensitivity; the report by Xu et al.^12^ showed that ΔCMRO_2_ is proportional to ΔpEtCO_2_, suggesting a dose-dependent effect of CO_2_ on CMRO_2_.^12^ Therefore, a sufficiently high ΔpEtCO_2_ should be attained to allow significant observations of CMRO_2_ reductions during hypercapnia. In addition, the suggested dose-dependency of CO_2_ on ΔCMRO_2_ would mean that discrepant ΔpEtCO_2_ values will lead to discrepant ΔCMRO_2_ findings. In line with this, discrepant findings on ΔCMRO_2_ during hypercapnia can also be caused by differences in the duration of the hypercapnic stimulus even though similar ΔpEtCO_2_ was reached. Future work on modulation of the duration of hypercapnic stimulus would shed light on this.

Assuming iso-metabolic challenges when performing calibrated BOLD experiments may lead to a systemic bias, as shown by previous simulation and experimental studies.^10,19,20^ A CO_2_-dependent reduction in CMRO_2_ (as has been shown in this work) would lead to an overestimation of the M-value and a concordant overestimation of the maximum possible BOLD signal change, which translates into an up to 50% overestimation in basal OEF.^19^ For activation-induced ΔCMRO_2_ estimates, one can expect a close to linear behavior between calibration bias and the overestimation of ΔCMRO_2_. This translates to a bias in the estimated OEF and CMRO_2_ changes (ΔCMRO_2_) during task-evoked calibrated BOLD studies. For example, Griffeth et al.^20^ (see Figure 8A in their report) showed that the bias in estimated ΔCMRO_2_ during activation was in the range of 5%–10% percentage point for 10%-15% reduction CMRO_2_ during hypercapnic calibration.

Further work by Blockley et al.^10^ and Merola et al.^19^ provide a sense of how large potential errors might be due to violation of the iso-metabolic assumption.^10,19^ The effect of a non-isometabolic stimulus on CVR experiments will depend on the type of technique used. As BOLD is sensitive to changes in deoxyhemoglobin; a decrease in CMRO_2_ during hypercapnia could lead to an overestimation of the BOLD response and thus in an overestimation of the BOLD CVR amplitude. The size of this potential bias in BOLD-CVR needs to be investigated further to see how clinically relevant this bias is. An approach to mitigate potential metabolic contamination using a hypercapnic stimulus in BOLD-CVR could be to add a hypoxic component to the gas mixture, as suggested by Peng et al.^17^ For ASL or ^15^O-H_2_O PET-based CVR studies, we do not expect a bias in CVR since these techniques are dominated by CBF changes and not sensitive to changes in deoxyhemoglobin.

The CMRO_2_ reported here under the room-air condition (144.5 ± 25.5 µmol/100 g/min) follows those previously reported based on TRUST-MRI, susceptibility-based Y_v_ measurements as well as the PET gold-standard.^46,54,61,62^ When ignoring the venous plasma O_2_ for the calculation of ΔCMRO_2,_ this results in a 0.7 percentage point increase for the difference in ΔCMRO_2_ between room-air and ‘CO_2_ in air’, the same increase was found for the difference between room-air and carbogen ΔCMRO_2_. Based on these calculations, venous plasma dissolved O_2_ played a negligible role in the arteriovenous oxygen difference, a key factor that determines CMRO_2_ under the conditions assessed herein. Specifically, for the carbogen condition, arterial plasma dissolved O_2_ was metabolized by the tissue (see Figure 3). Arterial plasma dissolved O_2_ plays a negligible role in the transportation of oxygen under physiological conditions. This is reflected by the small change in ΔCMRO_2_ when ignoring arterial and venous O_2_ for the ‘CO_2_ in air’ condition (−13.8% compared to −13.4% when including arterial and venous plasma dissolved O_2_). However, for carbogen, the estimated ΔCMRO_2_(%) drastically differed when ignoring the arterial and venous plasma dissolved O_2_ (−20.6% instead of −2.0%). Given the very high arterial plasma O_2_ values seen during carbogen, this difference was expected.

The setup we used for carbogen gas inhalation is a relatively simple way to evoke a vascular response and has seen use in numerous studies.^63–65^ Furthermore, carbogen has been reported to be more comfortable and possibly safer for patients and participants compared to a normoxic CO_2_ stimulus.^66^ Aside from its vasodilatory action, carbogen leads to a significant increase in arterial plasma dissolved O_2_. Similar to the debate surrounding the effect of hypercapnia on CMRO_2_, the question of whether changes in arterial O_2_ content modulate CMRO_2_ has also provided mixed conclusions. Studies looking at the potential neuroprotective effect of hyperoxia for acute treatment of traumatic brain injury have reported limited changes in CMRO_2_ using normobaric hyperoxia.^67^ This finding is supported by a physiological study measuring CMRO_2_ during prolonged apnea using trained free-divers performed by Ainslie et al.^68^ Interestingly, a significant post-treatment increase in CMRO_2_ of 32% was observed in severe traumatic brain injury patients when applying hyperbaric hypercapnia.^69^ In contrast, the work of Xu et al.,^13^ reported an ∼16.9% reduction in CMRO_2_ when breathing a normobaric fixed inspired O_2_ of 98%.^13^ In that study, a hypoxic stimulus was reported to increase CMRO_2_ providing the notion that varying inspired O_2_ content affects CMRO_2_ in a dose-dependent manner.^13^

In the context of our work, hyperoxia seems to affect the CMRO_2_. If hyperoxia had a neutral effect on CMRO_2_, the reduction in CMRO_2_ seen during the ‘CO_2_ in air’ condition would carry over to the carbogen condition, which was not observed. Similarly, a further decrease in CMRO_2_ would have exaggerated the negative effect of hypercapnia on CMRO_2_. Since neither of these responses was observed in our experiments, several hypotheses could explain our finding that CMRO_2_ did not significantly change during the carbogen condition. The addition of the hyperoxic component seems to mitigate the hypercapnia-induced reduction in CMRO_2._ Here, the addition of a hyperoxic component could have altered the brain arousal state and/or the neurophysiological reactions to the hypercapnic stimulus. The notion that an interplay between hypercapnic and hyperoxic states can modulate the brain’s response has been purported previously by Bain et al.^70^ It is important to note that while ‘CO_2_ in air’ induces a physiological condition that can be achieved naturally (i.e. being out of breath after running up several stairs, or breath-holding after hyperventilation), the effect of carbogen inhalation on blood gas composition is impossible to reach under normal circumstances. It stands to reason that accurately predicting the effect of hypercapnic-hyperoxic stimuli on CMRO_2_ is complex in a dynamic biological system as the human body, which has multiple mechanisms in place to actively maintain homeostasis.

### Considerations

Our observation of no significant differences in CBF between the ‘CO_2_ in air’ and carbogen conditions implies that the carbogen stimulus did not lead to significant O_2_-mediated vasoconstriction. This is in line with previously reported MRI findings and also supports results reported using PET.^26,45,71^ Hyperoxia is known to modulate arterial blood water T_1a_ due to hyperoxia; to account for this, we incorporated a global T_1,HO_ reduction in the CBF quantification as reported previously for the same carbogen stimulus,^34^ and investigated different T_1a_ scenarios (Supplementary Figure 3). It should be noted that intersubject variation will remain and will also depend on the subject's hematocrit. Any intersubject variation in CBF and arteriovenous O_2_ difference will translate directly to variation in CMRO_2_. From the sensitivity analysis on T_1_, we observed that increasing or decreasing the hyperoxic blood water T_1_ by 5%, CBF and CMRO_2_ increased by 4.1% or decreased by 4.7%, respectively (Supplementary Figure 2). Notably, the ΔCBF and ΔCMRO_2_ results did not significantly change for different T_1a_ scenarios using commonly used and recently reported T_1,NO_ and T_1,HO_ values (Supplementary Figure 3). It is our view that for this study, inter-subject variations were likely were ‘averaged out’, reducing the probability of systematic bias. It is important to emphasize that our results were derived from healthy subjects, using two commonly used stimuli. Based on the presented data, it would not be accurate to extrapolate results using different CO_2_ with O_2_ gas mixture concentrations, nor can we predict responses in patients with circulatory, pulmonary, or cerebrovascular pathologies. More research is needed to investigate whether changes in CMRO_2_ vary with different combinations of CO_2_ and O_2_ concentrations when using carbogen-based designs. How the respective responses might change in pathological situations remains an open question.

With regards to the Y_v_ measurement, the TRUST technique may exhibit a bias from hyperoxia-mediated changes in venous plasma T_2_. However, we observed that the venous plasma O_2_ content was negligible for all conditions (Figure 3), and thus could not influence the Y_v_ measurement for the carbogen condition. Furthermore, the TRUST technique relies on a single measurement taken at the occipital part of the superior sagittal sinus. While the superior sagittal sinus drains most of the total cerebral blood, it also drains the periosteum, skull, meninges, and CSF.^44^ Given that most of the blood flowing through the sinus is from the brain and that earlier reports on TRUST Y_v_ measurements at different locations have shown no difference in Y_v,_^72^ it is safe to presume the superior sagittal sinus as a representative location for a global Y_v_ measurement. In addition, we were mainly interested in the relative change of Y_v_ in the ΔCMRO_2_ comparison for the different conditions, for which the superior sagittal sinus is appropriate under the assumption that no redistribution of blood has occurred. There are other ways than pCASL to measure global CBF, such as phase-contrast MRI. These techniques have been previously compared and showed a close match.^55,73^

For diagnostics, treatment, or follow-up in patients, possible regional differences in CMRO_2_ might be of interest to clinicians. Our approach of measuring global Y_v_ excludes the possibility to examine regional differences in CMRO_2_. Regional Y_v_ measurements can be obtained using QSM-based techniques^74^ or other T_2_- based MRI acquisitions, like T_2_-relaxation-under-phase-contrast MRI,^75^ Velocity Selective Excitation and Arterial Nulling^76^ or by using techniques based on the Asymmetric Spin Echo.^77^ Such approaches could further benefit from subject-specific measures of Hct or the incorporation of local Hct measures.^78^ We assumed Hct values (0.42 for males, and 0.40 for females) as both the T_1_ and T_2_ values of blood depend on Hct. However, in healthy subjects, the Hct generally only varies by around 10%;^51^ it is unlikely that this would impact our Y_v_ results significantly. This could be different in disease though. An earlier reported sensitivity analysis on T_1_ differences on TRUST Y_v_ measurements demonstrated a negligible effect of slight differences in Hct on Y_v._^61^ For our study, the incorporation of subject-specific p_a_CO_2_ and (assumed) Hct in the model by Dash et al.^42^ did not impact the results dramatically (as shown in Figure 1(b)). Future (clinical) studies using such a physiological model will allow extraction of subject-specific blood oxygen content where information such as p_a_CO_2_, Hct, pH, and temperature can in principle be incorporated, depending on the study design and disease of interest.

## Conclusion

We found that a hypercapnic normoxic stimulus of 5% CO_2_ is not necessarily iso-metabolic to room-air but leads to a decrease in CMRO_2_ in healthy subjects. For a hypercapnic hyperoxic stimulus as carbogen, we demonstrate that it is more iso-metabolic to room-air. Although the oxygen saturation curve is dependent on p_a_CO_2_ (Bohr effect), amongst other parameters, correction for differences in blood p_a_CO_2_ did not significantly influence results. We believe these findings provide valuable insight into the global hemodynamic and metabolic effects of commonly used respiratory challenges. The reported findings can be useful for future experimental designs, BOLD signal modeling, and interpreting calibrated BOLD fMRI and CVR measurements using hypercapnic or hypercapnic-hyperoxic (carbogen) stimuli.

## Supplemental Material

sj-pdf-1-jcb-10.1177_0271678X211064572 - Supplemental material for Hemodynamic and metabolic changes during hypercapnia with normoxia and hyperoxia using pCASL and TRUST MRI in healthy adults Click here for additional data file. Supplemental material, sj-pdf-1-jcb-10.1177_0271678X211064572 for Hemodynamic and metabolic changes during hypercapnia with normoxia and hyperoxia using pCASL and TRUST MRI in healthy adults by Pieter T Deckers, Alex A Bhogal, Mathijs BJ Dijsselhof, Carlos C Faraco, Peiying Liu, Hanzhang Lu, Manus J Donahue and Jeroen C.W Siero in Journal of Cerebral Blood Flow & Metabolism
