# Supplementary material for: Hemodynamic and metabolic changes during hypercapnia with normoxia and hyperoxia using pCASL and TRUST MRI in healthy adults
Source: J Cereb Blood Flow Metab. 2021 Dec 1;42(5):861–75. doi: 10.1177/0271678X211064572 (PMC9014679; doi:10.1177/0271678X211064572)
Supplement: sj-pdf-1-jcb-10.1177_0271678X211064572 - Supplemental material for Hemodynamic and metabolic changes during hypercapnia with normoxia and hyperoxia using pCASL and TRUST MRI in healthy adults [file sj-pdf-1-jcb-10.1177_0271678X211064572.pdf]

Supplementary Material

White matter CBF correction for global CBF estimates for a single-compartment kinetic model

Simulations were performed to address the effect on white matter (WM) CBF quantification when using parameters for WM, tissue $T_{1,WM}$ and arterial (bolus) arrival time, BAT_{WM} instead of gray matter (GM) tissue $T_{1,GM}$ and BAT_{GM} in the ASL CBF quantification model as commonly done.

The conversion of pCASL measured ΔM to CBF can be considered as a scaling problem, when applying a single-compartment kinetic model (Buxton et al.[1]). Here, the scaling factor 'C' depends on the familiar tissue-specific parameters: T_1 and BAT and 'global' parameters: blood $T_{1,a}$, bolus duration τ, inversion efficiency α, blood/tissue water partition coefficient λ, and the voxel specific calibration $M_{0,b}$.
Note that the apparent T_1' dependency on CBF ($1/T_1' = 1/ T_{1,tissue} + f/\lambda$ with f = CBF in mL/g/s) in the kinetic model makes it deviate from a pure scaling problem, however, the effect of CBF on the apparent T_1 is negligible. The latter can be seen by the very slight broadening of the CBF normalized kinetic curve for different simulated CBF values with respect to inversion time TI (see Supplemental Figure 2 below).

A range of kinetic curves was generated for a range of CBF values (0 – 1.5 ml/g/s ≅ 0 – 90 mL/100 g/min) and subsequently normalized by the CBF value. Tissue specific values where $T_{1,GM}$ = 1.3s[2], $T_{1,WM}$ = 0.84s[2], BAT_{GM}[3] and BAT_{WM}[3].
The resulting, 'normalized kinetic curves', give the conversion from CBF to ΔM with respect to the inversion time TI. The scaling factor C, used to convert ΔM to CBF, is then obtained by taking the reciprocal of the normalized kinetic curve at the pCASL sequence's TI. In Supplemental Figure 2 below, CBF normalized kinetic curves for GM and WM based values, tissue T_1 and BAT, are depicted in blue and red, respectively. To convert measured ΔM to CBF (in mL/g/s) using GM based values one gets a scaling factor of $C_{GM} \approx 1/0.5 \approx 2$ for the pCASL sequence inversion time used here: TI = τ + PLD = 1.5s + 1.7s = 3.2s. To convert measured ΔM to CBF using white matter based values, one gets a scaling factor of $C_{WM} \approx 1/0.42 \approx 2.4$ at the same inversion time.

From this exercise, we observe that for WM regions, when using GM based values in CBF quantification, the CBF_{WM} is underestimated by a factor of $C_{WM}/C_{GM} \approx 2.4/2 \approx 1.2$, which is about 20%. This factor can be used to correct the CBF values in the WM ROI, i.e. increasing WM CBF by ~20%, yielding corrected global (whole-brain) CBF estimates and thus $CMRO_2$ estimates. We found that the $CMRO_2$ findings did not change notably when incorporating the WM correction on global CBF, the numerical values did change.

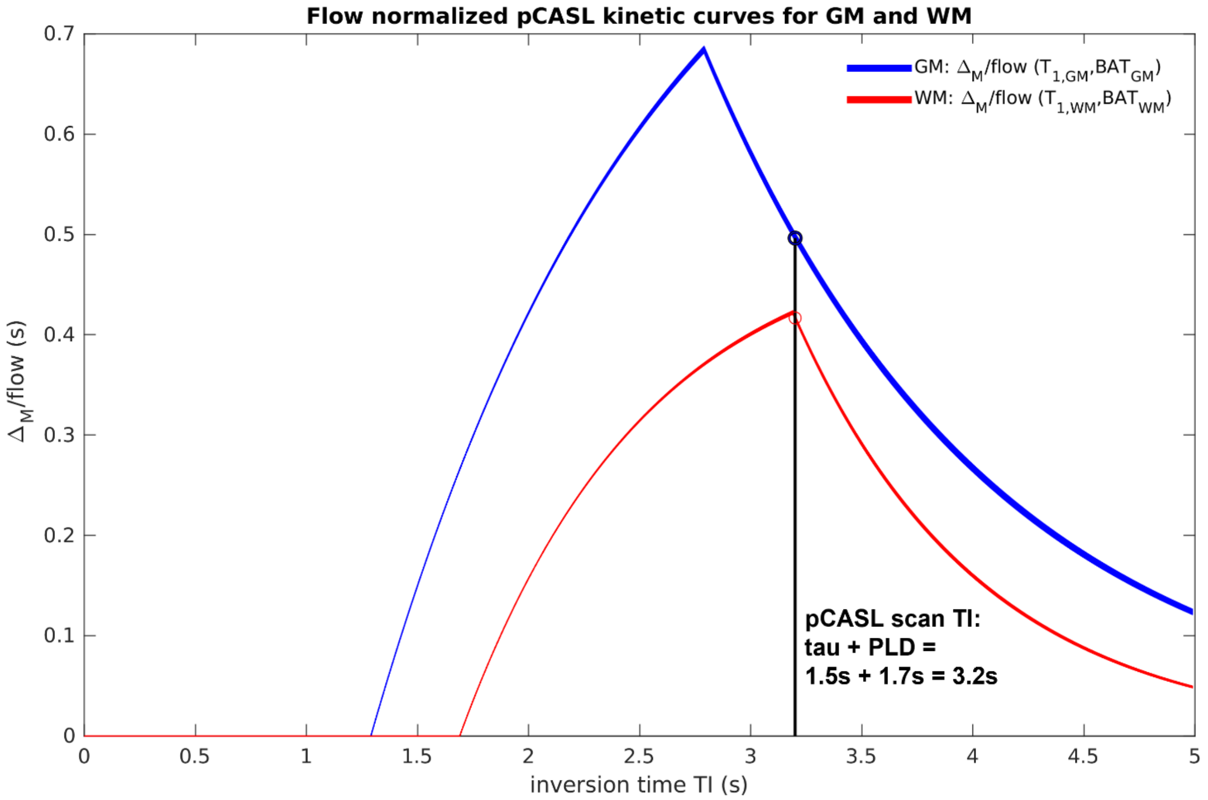

Supplementary Figure 1: Flow (CBF) normalized kinetic curves for GM and WM based values (tissue T1 and bolus arrival time, BAT) are depicted in blue and red, respectively. These curves give the conversion from CBF to ΔM with respect to the inversion time TI. The scaling factor C, to convert ΔM to CBF, is then obtained by taking the reciprocal of the normalized kinetic curve at the pCASL sequence's TI. To convert measured ΔM to CBF (in mL/g/s) using GM based values one gets a scaling factor of $C_{GM} \approx 1/0.5 \approx 2$ for the pCASL sequence inversion time used here: TI = τ + PLD = 1.5s + 1.7s = 3.2s. To convert measured ΔM to CBF using white matter based values, one gets a scaling factor of $C_{WM} \approx 1/0.42 \approx 2.4$ at the same inversion time. As a result, when using GM based values in CBF quantification, the WM CBF is underestimated by a factor of $C_{WM}/C_{GM} \approx 2.4/2 \approx 1.2$, which is about 20%. This factor can be used to correct the CBF values in the WM ROI, i.e. increasing WM CBF by ~20%, yielding corrected global (whole-brain) CBF estimates and thus $CMRO_2$ estimates.

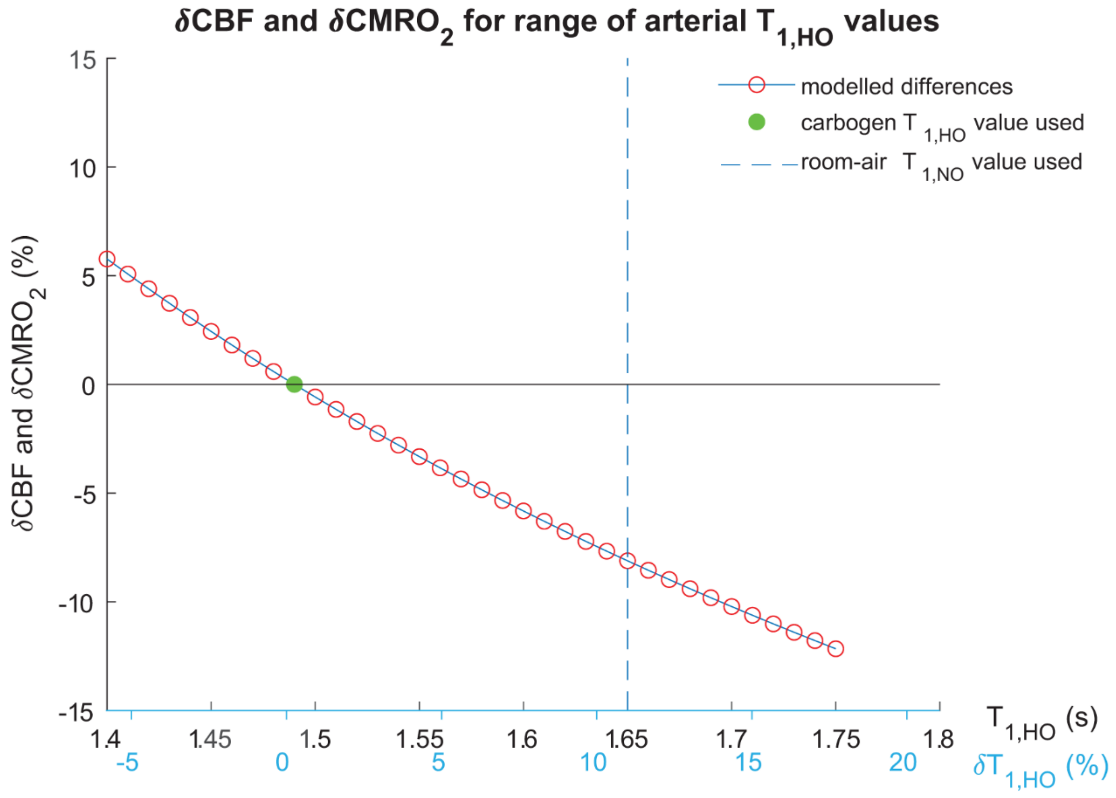

Supplementary Figure 2. Sensitivity analysis of the effect on hyperoxic arterial blood water $T_{1,HO}$ on CBF and $CMRO_2$ quantification. The $T_{1,HO}$ value used for carbogen (1.49s) and variations therein (δ) were plotted against the modelled percentage change (δ) in CBF and $CMRO_2$ quantification. The used $T_{1,NO}$ for room-air (1.65s) is depicted by the dashed blue line. Note CBF and $CMRO_2$ are directly proportional by the arteriovenous O_2 difference. The resulting percentage change in CBF and $CMRO_2$ were plotted for a range of possible $T_{1,HO}$ values, for absolute values and the percentage difference with respect to the reference $T_{1,HO}$ (=1.65s) for carbogen. CBF = cerebral blood flow; $CMRO_2$ = cerebral metabolic rate of oxygen, $T_{1,HO}$ = arterial blood water T_{1a} during hyperoxia, $T_{1,NO}$ = arterial blood water T_{1a} during normoxia.

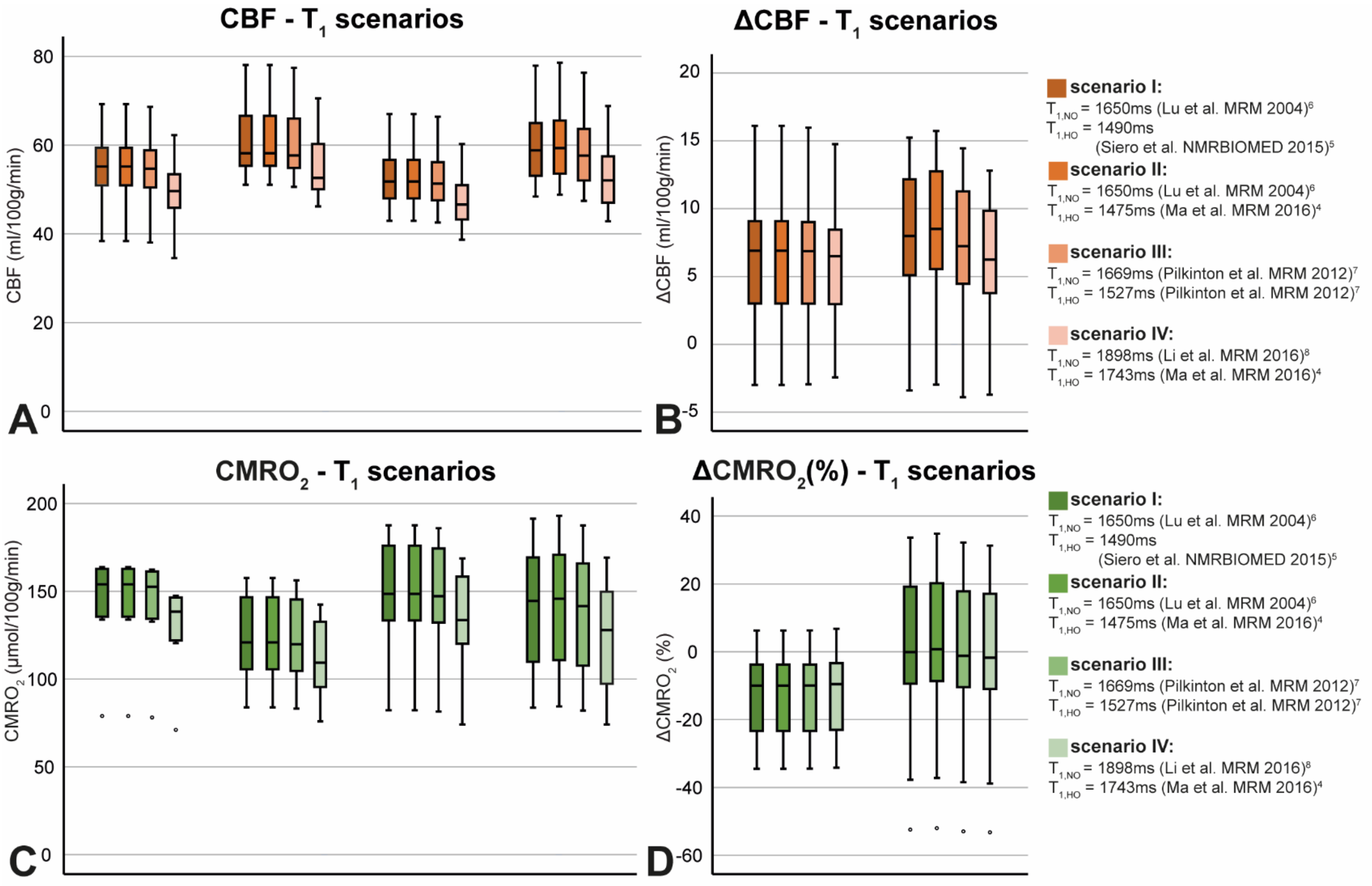

Supplementary Figure 3. A) CBF, **B)** ΔCBF, **C)** $CMRO_2$ and **D)** $\Delta CMRO_2$ results for different arterial blood water T_{1a} scenarios for normoxic ($T_{1,NO}$) and hyperoxic ($T_{1,HO}$) conditions. Although the absolute CBF and $CMRO_2$ results change in value, the impact on the ΔCBF and notably the $\Delta CMRO_2$ changes, which is the topic of this study, did not change significantly for the different T_1 scenarios. For the hyperoxic (carbogen) condition, a p_aO_2 of 460 mmHg was assumed, and for scenarios II and IV we incorporated the reported hyperoxic T_{1a} relativity by Ma et al.[4] (see Methods 'Effect of arterial blood water T_{1a} on CBF and $CMRO_2$ quantification'. CBF = cerebral blood flow; $CMRO_2$ = cerebral metabolic rate of oxygen; p_aO_2 = partial pressure of arterial O_2, $T_{1,HO}$ = arterial blood water T_{1a} during hyperoxia, $T_{1,NO}$ = arterial blood water T_{1a} during normoxia. The boxplots show the minimum, maximum, median and interquartile range, open circles denote outliers.

Supplementary Table 1. Cerebral blood flow (CBF) in gray matter, venous oxygenation (Y_v), oxygen extraction fraction (OEF), and cerebral metabolic rate of oxygen ($CMRO_2$) results for the room air, 'CO_2 in air' and carbogen breathing conditions for all subjects.

	**cerebral blood flow** CBF (ml/100g/min)				**venous oxygenation** Y_v (%)				**oxygen extraction fraction** OEF				**cerebral metabolic rate of O_2** $CMRO_2$ (μmol/100g/min)			
**subj. nr**	**room-air1**	**CO_2 in air**	**room-air2**	**carbogen**	**room-air1**	**CO_2 in air**	**room-air2**	**carbogen**	**room-air1**	**CO_2 in air**	**room-air2**	**carbogen**	**room-air1**	**CO_2 in air**	**room-air2**	**carbogen**
1	59.35	67.66	42.94	57.75	70.00	79.00	68.00	84.50	0.29	0.19	0.31	0.20	143.05	108.43	111.53	107.56
2	51.34	55.33	49.58	59.46	68.00	71.00	67.00	73.50	0.31	0.28	0.32	0.31	133.92	128.93	133.46	169.33
3	50.94	51.04	48.00	53.09	63.00	72.50	64.00	73.00	0.36	0.26	0.35	0.31	155.62	112.89	142.45	153.56
4	50.12	57.30	52.45	49.07	63.00	68.50	65.00	70.00	0.36	0.30	0.34	0.34	153.17	146.65	150.92	154.93
5	38.37	54.47	45.55	48.39	73.00	79.00	76.00	79.00	0.26	0.19	0.22	0.26	78.99	83.92	82.26	109.92
6	69.25	78.06	66.99	77.90	71.00	76.00	66.00	88.50	0.28	0.22	0.33	0.16	163.22	148.55	187.66	116.85
7	53.89	56.91	51.13	56.95	63.00	66.00	56.00	72.00	0.36	0.33	0.43	0.32	163.84	157.61	187.33	169.75
8	56.87	63.49	52.56	64.95	65.00	71.50	66.00	82.00	0.34	0.27	0.33	0.23	162.83	144.93	146.17	135.53
9	62.03	59.04	56.65	68.81	68.00	77.00	64.00	73.00	0.31	0.21	0.35	0.32	154.81	101.43	160.64	191.44
10	56.42	66.58	57.20	63.29	69.00	78.50	61.00	90.00	0.30	0.20	0.38	0.15	135.60	105.60	176.05	83.77

**mean**	**54.86**	**60.99**	**52.31**	**59.97**	**67.30**	**73.90**	**65.30**	**78.55**	**0.32**	**0.25**	**0.34**	**0.26**	**144.51**	**123.89**	**147.85**	**139.26**
std	8.22	8.03	6.83	9.13	3.62	4.65	5.10	7.31	0.04	0.05	0.05	0.07	25.50	24.78	33.29	34.10

std = standard deviation

Supplementary Table 2. The change in end-tidal CO_2 ($pEtCO_2$), global cerebral blood flow (CBF), venous oxygenation (Y_v), oxygen extraction fraction (OEF), and cerebral metabolic rate of oxygen ($CMRO_2$) for the 'CO_2 in air' and carbogen breathing conditions with respect to the room air breathing condition for all subjects.

	**change in $pEtCO_2$**		**change in CBF**				**change in Y_v**		**change in OEF**				**change in $CMRO_2$**			
	**$\Delta pEtCO_2$ (mmHg)**		**ΔCBF (ml/100g/min)**		**ΔCBF (%)**		**ΔY_v (%)†**		**ΔOEF**		**ΔOEF (%)**		**$\Delta CMRO_2$ (µmol/100g/min)**		**$\Delta CMRO_2$ (%)**	
**subj. nr**	**CO_2 in air**	**carbogen**	**CO_2 in air**	**carbogen**	**CO_2 in air**	**carbogen**	**CO_2 in air**	**carbogen**	**CO_2 in air**	**carbogen**	**CO_2 in air**	**carbogen**	**CO_2 in air**	**carbogen**	**CO_2 in air**	**carbogen**
1	3.00	4.20	8.31	14.81	12.28	25.65	11.39	19.53	-0.10	-0.10	-50.28	-51.63	-34.62	-3.97	-24.20	-3.56
2	5.70	1.00	3.99	9.88	7.22	16.61	4.23	8.84	-0.03	-0.01	-11.78	-2.69	-4.99	35.88	-3.73	26.88
3	2.00	0.80	0.10	5.10	0.20	9.60	13.10	12.33	-0.10	-0.04	-38.06	-11.33	-42.73	11.12	-27.46	7.80
4	5.10	3.70	7.19	-3.39	12.54	-6.90	8.03	7.14	-0.06	0.00	-19.27	1.10	-6.52	4.01	-4.26	2.66
5	3.30	5.20	16.10	2.84	29.55	5.86	7.59	3.80	-0.06	0.03	-33.50	13.39	4.93	27.67	6.24	33.64
6	4.30	4.20	8.81	10.91	11.29	14.00	6.58	25.42	-0.05	-0.17	-23.72	-102.39	-14.66	-70.80	-8.98	-37.73
7	2.60	6.60	3.01	5.82	5.30	10.21	4.55	22.22	-0.03	-0.11	-9.69	-33.45	-6.23	-17.57	-3.80	-9.38
8	3.00	5.80	6.62	12.39	10.43	19.07	9.09	19.51	-0.07	-0.10	-25.34	-44.77	-17.90	-10.63	-10.99	-7.28
9	7.40	7.00	-2.99	12.16	-5.07	17.67	11.69	12.33	-0.10	-0.03	-44.98	-10.82	-53.37	30.81	-34.48	19.18
10	5.50	6.00	10.17	6.09	15.27	9.62	12.10	32.22	-0.10	-0.23	-51.34	-153.09	-30.01	-92.28	-22.13	-52.42
**mean**	**4.19**	**4.45**	**6.13**	**7.66**	**9.90**	**12.14**	**8.84**	**16.33**	**-0.07**	**-0.08**	**-30.80**	**-39.57**	**-20.61**	**-8.58**	**-13.38**	**-2.02**
std	1.70	2.16	5.40	5.45	9.29	8.84	3.17	8.94	0.03	0.08	15.21	52.16	18.83	42.65	12.98	27.03

$pEtCO_2$ = end-tidal partial pressure of CO_2, CBF = global cerebral blood flow, Y_v = venous blood oxygenation, OEF = oxygen extraction fraction, $CMRO_2$ = cerebral metabolic rate of oxygen, std = standard deviation, †note this is the fractional change in percentage in Y_v, i.e. not percentage points.

References

1. Buxton RB, Frank LR, Wong EC, et al. A general kinetic model for quantitative perfusion imaging with arterial spin labeling. *Magnetic Resonance in Medicine* 1998; 40: 383–396.

2. Rooney WD, Johnson G, Li X, et al. Magnetic field and tissue dependencies of human brain longitudinal 1H2O relaxation in vivo. *Magnetic Resonance in Medicine* 2007; 57: 308–318.

3. Juttukonda MR, Li B, Almaktoum R, et al. Characterizing cerebral hemodynamics across the adult lifespan with arterial spin labeling MRI data from the Human Connectome Project-Aging. *NeuroImage*; 230. Epub ahead of print 2021. DOI: 10.1016/j.neuroimage.2021.117807.

4. Ma Y, Berman AJL, Pike GB. The effect of dissolved oxygen on the relaxation rates of blood plasma: Implications for hyperoxia calibrated BOLD. *Magnetic Resonance in Medicine* 2016; 76: 1905–1911.

5. Siero JCW, Strother MK, Faraco CC, et al. In vivo quantification of hyperoxic arterial blood water T1. *NMR in Biomedicine* 2015; 28: 1518–1525.

6. Lu H, Clingman C, Golay X, et al. Determining the longitudinal relaxation time (T1) of blood at 3.0 tesla. *Magnetic Resonance in Medicine* 2004; 52: 679–682.

7. Pilkinton DT, Hiraki T, Detre JA, et al. Absolute cerebral blood flow quantification with pulsed arterial spin labeling during hyperoxia corrected with the simultaneous measurement of the longitudinal relaxation time of arterial blood. *Magnetic Resonance in Medicine* 2012; 67: 1556–1565.

8. Li W, Grgac K, Huang A, et al. Quantitative theory for the longitudinal relaxation time of blood water. *Magnetic Resonance in Medicine* 2016; 76: 270–281.
